# Real-world effects of hyoscine butylbromide combined with paracetamol in women with dysmenorrhea: a patient survey

**DOI:** 10.3389/fmed.2025.1581059

**Published:** 2025-04-14

**Authors:** Kathrin Stewen, Selina Miehle, Harald Weigmann, Roxana Schwab

**Affiliations:** ^1^Department of Obstetrics and Gynecology, University Medical Center of Johannes Gutenberg University, Mainz, Germany; ^2^Sanofi, Frankfurt am Main, Germany

**Keywords:** dysmenorrhea, hyoscine butylbromide, paracetamol, fixed-dose combination, pharmacy-based patient survey, real-world evidence

## Abstract

**Introduction:**

Dysmenorrhea symptoms are frequent and often self-treated using non-prescription medicines.

**Methods:**

To further characterize women with dysmenorrhea using a combination of hyoscine butylbromide plus paracetamol (PLUS) for self-management of their complaints, we performed a secondary analysis of a published pharmacy-based patient survey.

**Results:**

A total of 314 women (mean age: 32.3 years) with dysmenorrhea reported a pain and cramps intensity of 7.45 ± 2.13 (means ± SD) on a 0–10 Likert scale, which was reduced to 2.86 ± 1.81 upon treatment. Associated impairments of work/daily chores, leisure activities, and sleep were improved by 64.6, 62.2, and 70.4%, respectively. The onset of symptom relief was within 60 min in 84.7%. Tolerability was rated as very good or good by 97.2%; 82.8% were repeat users, 97.5% reported their intention to purchase the product again, and 97.1% reported their intention to recommend it to relatives, friends, and colleagues.

**Discussion:**

These findings confirm the efficacy and tolerability data on PLUS from randomized controlled trials in a larger group of women conducting self-management of their dysmenorrhea in a real-world setting. Future studies should compare PLUS to other non-prescription treatments.

## 1 Introduction

Dysmenorrhea is a common menstrual complaint affecting 16–91% of women of reproductive age (1). This wide range of prevalence may be attributable to differences in definition, sampling methods, and populations being investigated. It can be a symptom of endometriosis (secondary dysmenorrhea), and various risk factors other than endometriosis have been identified including heavy menstrual loss, premenstrual symptoms, irregular menstrual cycles, aged younger than 30 years, clinically suspected pelvic inflammatory disease, sexual abuse, menarche before 12 years of age, low body mass index, smoking, and sterilization (1–3). The underlying pathophysiology is not fully clear but at least for primary endometriosis may involve increased formation of prostaglandins that leads to vasoconstriction followed by the release of anaerobic metabolites that stimulate pain receptors (4). Dysmenorrhea is typically described as a cramping pain in the lower abdomen starting at the onset of menstrual flow and often lasting 8–72 h with severe dysmenorrhea-associated pain present in 2–29% (1). Many women report the occurrence of pain during each period (5). Frequent accompanying symptoms include nausea, vomiting, diarrhea, headache, muscle cramps, lower back pain, fatigue, and, particularly in cases of severe dysmenorrhea, sleep disturbance (2, 3). Thus, dysmenorrhea is one of the most common causes of pelvic pain and has an adverse impact on quality of life, work productivity, absenteeism, and healthcare utilization (1–3, 6, 7).

Guideline-recommended medical management options include non-steroidal anti-inflammatory drugs and hormonal contraceptives (2, 3), considered to act symptomatically and interfering with menstrual cycle-related hormonal changes, respectively. While hormonal contraceptives are prescription drugs in most countries, non-steroidal anti-inflammatory drugs are often available without a prescription as over-the-counter (OTC) products enabling women to self-manage their condition. Recent reviews in the field support empiric treatment without additional testing (4). Accordingly, 36–70% of women manage their dysmenorrhea by self-medication often involving mefenamic acid, ibuprofen, or paracetamol (8, 9).

The anti-spasmodic hyoscine butylbromide (HBB) (10) is efficacious in a mouse model of dysmenorrhea (11). Following several open-label studies in women with dysmenorrhea applying HBB alone or in combination with metamizole or with lysine clonixinate (12–14), three controlled trials were reported. HBB (40 mg b.i.d.) had comparable efficacy relative to aspirin (300 mg b.i.d.) and greater efficacy than placebo in a double-blind crossover study in 20 women (15). A fixed-dose combination of 10 mg HBB plus 500 mg paracetamol (PLUS) had superior efficacy to both placebo and HBB alone in a mixed group of 45 patients with abdominal pain, including women with dysmenorrhea in a double-blind randomized controlled trial (RCT) (16). Most importantly, a double-blind, placebo-controlled, crossover RCT in 125 women with dysmenorrhea found that PLUS and lysine clonixinate plus propinox starting 3 days before onset of menses and lasting for 5 days thereafter found that both active treatments reduced pain relative to placebo with PLUS numerically causing the greatest reduction (17). Accordingly, PLUS is approved for the treatment of dysmenorrhea-associated cramps and pain in 10 countries in Europe, Asia, and Latin America. However, HBB or PLUS is not explicitly mentioned in relevant guidelines (2, 3), and the overall knowledge on which women choose to self-manage dysmenorrhea with PLUS and their experience is limited.

Pharmacy-based patient surveys (PBPS) are an established tool to obtain real-world evidence on OTC medications (18–22). A recent PBPS evaluated three preparations for the treatment of abdominal cramps and pain in 1686 patients (23); this included 329 women reporting the use of PLUS for dysmenorrhea. Here, we characterize these women and the self-reported effect of PLUS in an exploratory, *post-hoc* analysis.

## 2 Patients and methods

Details of study design and tested products have been reported (23). Briefly, a non-interventional, prospective PBPS was performed among 1,686 patients who had purchased an OTC product for the self-management of abdominal cramps and pain. This included 641 patients using PLUS (Buscopan plus^®^) providing information about baseline symptoms and their changes following intake of the first dose of PLUS. The questionnaire included a question on the indication for which PLUS was used with the options of “abdominal cramps and pain,” “urinary complaints such as urinary tract infection,” “dysmenorrhea,” and “other” with multiple nominations being possible. This manuscript focuses on the subgroup of 329 women reporting to have used PLUS for the indication of dysmenorrhea, regardless of additional indications being stated.

Inclusion criteria of the primary study (23) were the purchase of the product, willingness and ability to fill out the paper-based questionnaire, and an age of ≥18 years. There were no prespecified exclusion criteria in the main study. However, we excluded 13 women from the analysis because they had either an age < 18 years (*n* = 3) or > 51 years (n = 10; *post-hoc* decision). The three women who were < 18 years were excluded because of not meeting the inclusion criteria and the 10 women who were >51 years because we considered their age as implausible regarding the self-reported diagnosis of dysmenorrhea based on an upper end of 51 years for the 95% confidence interval for onset of menopause (24); an additional two women did not provide age data, leaving 314 women for analysis. In cases where answers to one or more questions were missing, a participant was excluded from those questions (up to 3 patients per question) but not for the overall analysis.

The survey was anonymous, and no information was collected allowing *post-hoc* identification or contact of the participants. Applicable German laws and regulations neither required nor recommended the involvement of an ethical committee for an anonymous survey at the time when it was conducted; this is in line with other recently reported PBPS from Germany (18–22). Based on the anonymity of the survey, returning of the questionnaire was considered as consent to participate.

The questionnaire included questions in the following categories:

- demographic variables (gender, age),
- number of days with symptoms in the past 30 days,
- the perceived trigger of the current complaints (selection from a list of possible triggers),
- the time span of pain/complaints before treatment initiation, that is, proposing the categories “first signs of a ‘bad day’,” “directly” and 30 min, 60 min, 2 h, or more than 2 h after onset of symptoms.
- The current condition was described by asking questions on the intensity of pain/complaints prior to first ingestion of the medication and on their impact on work/daily chores, leisure activities, and sleep.
- The global efficacy and tolerability were rated on a categorical scale of very good, good, moderate, and poor.

The questions on the severity of pain/complaints and on associated impact were rated on an 11-point Likert scale (0–10 from “no” to “very strong pain/complaint/impact”); all other questions were asked categorically. The questions on the intensity of pain/complaint and the impact of the current condition were repeated after drug intake. The questionnaires asked about the time to onset of relief following first ingestion with available options of 0–5, 6–15, 16–30, 31–45, 46–60, and >60 min. Finally, the perceived general effectiveness, tolerability, and treatment satisfaction were captured.

Data are reported as means ± SD and as medians with interquartile range (IQR) for continuous parameters and in absolute numbers and % of total for categorical parameters. All analyses reported in the primary publication have currently been performed for the women reporting the use of the product for the treatment of dysmenorrhea symptoms. In line with recent guidelines for enhanced robustness of data analysis (25, 26), we considered all data to be exploratory, that is, not testing a prespecified statistical null hypothesis; inherently, a *post-hoc* analysis cannot be hypothesis testing as that would require a random sample. Therefore, as recommended by leading statisticians (27, 28), we do not report *p*-values and focus on effect sizes with the presentation of 95% CI.

## 3 Results

### 3.1 Demographics and baseline patient characteristics

The participants had a mean age of 32.3 ± 9.2 years (IQR 25–40 years). The largest fraction obtained PLUS based on a recommendation by a pharmacist (46.2%), followed by recommendations by relatives or friends (34.1%) or by a physician (18.5%); less frequent reasons were advertisements on TV (12.4%), information on the internet (8.0%), and social media (6.1%); 15.3% did not provide a specific reason. Similar fractions of users had obtained PLUS in a stationary pharmacy (52.5%) and in an online pharmacy (47.5%). Interestingly, 15.3 and 82.8% reported to be first and repeat users, respectively.

Concomitant abdominal cramps and pain, urinary tract complaints, and “other” were reported by 98, 18, and 11 women (31.2, 5.7, and 3.5%, respectively). The mean and median number of days within the past month with such concomitant complaints was 4.34–4.54 and 2–3, respectively (Table 1). Of note, data on bloating and flatulence are difficult to interpret as 90 and 102 women, respectively, had missing data for these parameters; 82.8% of participants reported being repeat users of PLUS. The latter data had been captured in the underlying study (23); they are reported here for completeness only. We consider missing data on these two parameters irrelevant as neither is a symptom of dysmenorrhea.

**Table 1:** Concomitant complaints of eligible survey participants in the past 30 days.

Concomitant complaint	Incidence (number of days/month)
Complaints in the past 30 days	
Cramps and pain	4.51 ± 3.97 (3; 2–5)
Bloating	4.34 ± 5.42 (3; 0–6)
Flatulence	4.54 ± 6.03 (2; 0–7)

Data are shown as means ± SD of number of days with median and interquartile range in parentheses. Multiple nominations were possible for concomitant complaints.

**Figure 1:**
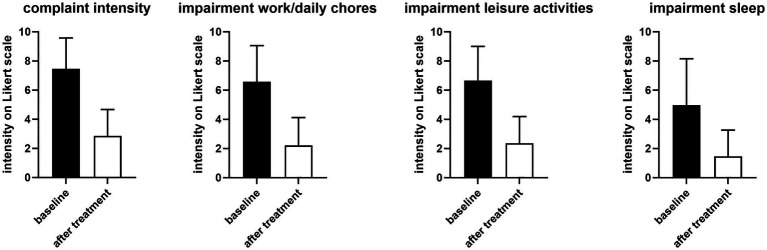
Intensity of overall complaints and impairment of work/daily chores, leisure, and sleep before (filled bars) and 1 h after first dose of treatment (open bars). Data are shown as means ± SD. The before vs. after difference was −4.59 [−4.90; −4.28] for complaint intensity, −4.37 [−4.72; −4.02] for impairment of work/daily chores, −4.29 [−4.62; −3.96] for impairment of leisure activities, and −3.51 [−3.92; −3.11] for impairment of sleep.

On an 11-point (0–10) Likert scale, the mean and median intensity of overall complaints before treatment were 7.45 ± 2.13 and 8 (IQR 7–9), respectively (Figure 1). This was associated with impairments of work/daily chores, leisure activity of comparable intensity and, to a somewhat smaller extent, of sleep (Figure 1).

Participants were asked to identify perceived triggers from a list with multiple possible options. The most frequently reported trigger was “other” (*n* = 191, 60.8%), most likely reflecting that the menstrual cycle had not been mentioned in the list because the underlying study had a much broader focus on abdominal cramps and pain (Table 2). Perceived triggers reported by at least 10% of participants included stress in general, bloating, and nutrition (too fatty or too sweet).

**Table 2:** Perceived triggers of current complaints as selected from a list being provided with multiple nominations possible.

Trigger	Incidence (%)
Stress	80 (25.3)
Bloating	57 (18.0)
Nutrition	45 (14.2)
Too little physical activity	30 (9.5)
Food intolerance	30 (9.5)
Sluggish bowels	28 (8.9)
Constipation	27 (8.5)
Flatulence	25 (7.9)
Diarrhea	18 (5.7)
Unfavorable intestinal bacteria	14 (4.4)
Infection	12 (3.8)
Heartburn	9 (2.8)
Environmental pollution	4 (1.3)
Other	193 (61.1)
Unknown	29 (9.2)

Data are shown as absolute numbers and as % of participants in parentheses.

### 3.2 Treatment responses

**Figure 2:**
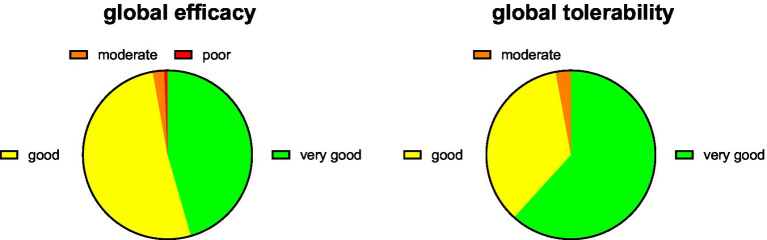
Global efficacy and tolerability rating. Data are shown as % of participants providing one of the four possible ratings.

Treatment with PLUS markedly reduced the intensity of complaints and impairment of work/daily chore and leisure activities and of sleep (Figure 1). Thus, the intraindividual mean reduction of complaint intensity on the 0–10 Likert scale was 4.55 ± 2.63 (median reduction 5). The improvement of impairment of work/daily chores and leisure activities was >60% and that of sleep approximately 70%. Accordingly, 45.5 and 51.6% rated the global efficacy of PLUS as very good or good, respectively (Figure 2). Similarly, 61.8 and 35.4% rated global tolerability as very good or good, respectively (Figure 2). Of note, none of the participating women rated global tolerability as poor. In line with this experience 97.5% reported their intention to purchase the product again, and 97.1% reported their intention to recommend it to relatives, friends, and colleagues.

**Figure 3:**
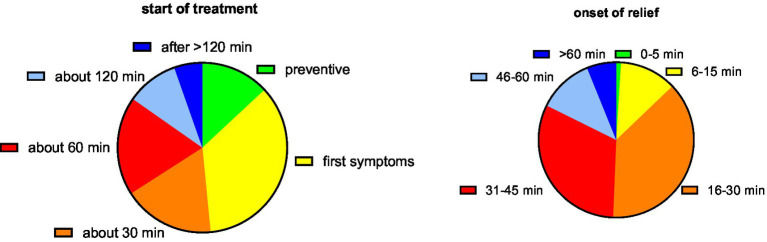
Start of treatment relative to the onset of symptoms and onset of symptom relief relative to tablet ingestion. Data are shown as % of participants providing one of the six possible ratings.

The participants used the first tablet at different times relative to the onset of symptoms (Figure 3). While the largest group (35.4%) reported initiating treatment upon emergence of first symptoms, similarly sized groups started 30 and 60 min after onset, and some even as late as after 2 h; interestingly, 13.1% of women used PLUS prior to onset of symptoms when they experienced the first signs of having a bad day. The self-reported onset of symptom relief after ingestion of the first dose of medication was after 30 min or earlier in 50.4, and 93.3% reported after less than 60 min (Figure 3).

## 4 Discussion

PLUS is an approved medication for the treatment of dysmenorrhea in at least 10 countries. While the effects of HBB and paracetamol against cramps and pain are well documented (10), the available evidence in the public domain underlying use of the combination in dysmenorrhea is limited; it rests on three open-label studies (12–14) followed by one RCT (17). Therefore, we have used data from a recently reported PBPS that had included many women using PLUS for the treatment of dysmenorrhea symptoms (23) to better characterize these women and the perceived treatment benefit.

### 4.1 Critique of methods

The present manuscript reports a *post-hoc* analysis of the subgroup of women reporting to have used PLUS for the treatment of dysmenorrhea. This subgroup was taken from an overall analysis primarily based on the pooled group of participants irrespective of indication (23). The primary study covered three preparations (PLUS, HBB as monotherapy, and peppermint oil) approved for use in multiple indications with abdominal cramps and pain as a shared characteristic. Therefore, it had primarily been designed around gastrointestinal symptoms (23) and no specific questions about dysmenorrhea were asked. Moreover, no separation between primary and secondary dysmenorrhea (due to endometriosis) was made. While this is a study limitation, the present data represent the largest cohort of women using PLUS as a self-medication product to treat dysmenorrhea symptoms; therefore, it provides relevant insight to characterize these women and their efficacy and tolerability experience.

The underlying study is a PBPS, which implies inherent weaknesses and strengths. PBPS and other non-interventional studies are unsuitable to test the effectiveness and safety of a medication relative to an established comparator such as a placebo. Therefore, no claims on absolute efficacy and safety should be derived from PBPS because RCTs should address those; possible contributions of a placebo component to the efficacy data cannot be excluded. Presently, only limited data from RCTs support the use of PLUS in the treatment of dysmenorrhea (17), but based on mechanistic plausibility (11) and on the established efficacy and safety of HBB and paracetamol (10) as components of PLUS, at least 10 countries in Europe, Asia, and Latin America have approved the use of PLUS for the treatment of dysmenorrhea.

Inherent limitations of PBPS include the reliance on self-reported data, a possible recall bias, and the lack of a control group. On the other hand, RCTs also have limitations: They have rigid inclusion and exclusion criteria often resulting in a group of participants that does not match those using the product in real life. Therefore, particularly in OTC products, PBPS have become an established tool to evaluate such products in real-world settings. This includes indications such as functional bowel disorders (22, 23), headache and migraine (18, 21, 29), and cough and cold (19, 20). These strengths and limitations inherent to PBPS should be considered in interpreting the present data.

In line with recommendations from leading statisticians (27, 28), the underlying main study had been designed as exploratory and did not address a pre-specified statistical null hypothesis. The *post-hoc* nature of the present analyses further contributes to an exploratory character. Therefore, no statistical null hypothesis testing was performed as in the underlying primary study.

### 4.2 Characterization of women using PLUS for the treatment of dysmenorrhea symptoms

Our observation that almost two thirds of women reported having chosen PLUS based upon the recommendation of a physician or pharmacist indicates its widespread acceptance by healthcare professionals in countries where it is available. However, little is known about the characteristics of users of this product in a self-management setting. Our cohort of 314 women had a mean age of 32.3 years, which is slightly older than that in the RCT (26.4 years) (17). The latter is also in line with the epidemiological observation that dysmenorrhea is predominantly a condition of younger women (1). Prior study had shown that the intensity of pain typically is moderate to severe. For example, a survey among 500 college students found a mean pain intensity of 5.0 on a 10-point visual analog scale (5) compared to 7.5 on an 11-point Likert scale in our survey. This difference may reflect that women with dysmenorrhea seeking medical treatment are likely to have more severe symptoms than those reporting the presence of the condition in an epidemiological study and not necessarily seeking treatment. An additional possible explanation is that women using an OTC product for the self-management of dysmenorrhea symptoms may be older because this group may have started self-management only after having consulted a physician, that is, have a more extended disease history. This idea is supported by the observation that 18.5% reported having chosen to use PLUS based upon the recommendation of a physician (and another 46.2% of a pharmacist), implying that they had already consulted a physician about their dysmenorrhea.

While dysmenorrhea mainly manifests as abdominal cramps and pain, it can be accompanied by other symptoms including sleep disturbance; it often leads to impaired quality of life and work productivity (1–3). Our survey data reflect that with a reported degree of impairment of work/daily chores, leisure activities, and sleep of 6.6, 6.6, and 5.0 on an 11-point Likert scale (Figure 1). Other known accompanying symptoms such as constipation and diarrhea, or factors perceived as triggers, such as too little physical activity (1–3) were also frequently present in our survey (Table 2). Several risk factors for dysmenorrhea have been identified, including a high stress level (1–3). Stress (25.3%) was the second most often mentioned trigger in our survey. As the underlying study was primarily designed to explore abdominal cramps and pain in general, our question on perceived triggers did not include a specific option on menstruation; the 61.1% reporting “other” as trigger can be interpreted to reflect menstruation as the primary trigger.

### 4.3 Outcomes in women using PLUS for the treatment of dysmenorrhea symptoms

The only available RCT of PLUS in women with dysmenorrhea reported a reduction of pain intensity by 47% (from 2.72 ± 0.6 to 1.45 ± 0.87 on a scale from 0 to 4) (17). The decrease in complaint intensity in the present PBPS was 62% (from 7.45 ± 2.13 to 2.86 ± 1.81 on a scale from 0 to 10). Greater effects in open-label studies than in blinded RCTs are frequent in clinical medicine. Importantly, the effect of PLUS was greater than with placebo (32% reduction in pain) in the RCT, which also manifested as a greater percentage of women reporting improved symptoms with PLUS than with placebo (82% vs. 63%) (17). PLUS also reduced concomitant headaches, palpitations, diarrhea, breast pain, and general discomfort in the RCT. The present data expand this knowledge by showing that the cramps and pain and the resulting impairments of work/daily chores, leisure activities, and sleep are similarly improved. Accordingly, 97.1% of participants in the PBPS rated global efficiency as very good or good (Figure 2).

While dysmenorrhea pain abates naturally over several days, the RCT showed that PLUS reduces pain not only on the first day of treatment but also thereafter (17). Our data expand this observation by demonstrating that onset of symptom relief occurs within less than an hour in 84.7% of women and in 50.4% even within 30 min (Figure 3). A rapid onset of symptom relief is important to patients; this finding merits a more detailed investigation in a controlled study.

The incidence of adverse events did not differ between PLUS and placebo in the RCT (17). Similarly, 97.2% of women in the present study rated global tolerability as very good or good (Figure 2). No specific adverse events were reported, but we cannot exclude an underreporting of adverse events in PBPS.

Taken together, these data show that women selecting self-management of their dysmenorrhea symptoms by an OTC product such as PLUS are slightly older than those in epidemiological or controlled studies and reported a greater pain intensity than epidemiological studies. Despite being older, which implies a longer disease history, the degree of symptom relief in using PLUS in an OTC setting caused symptom relief that was at least as large if not greater than reported in the RCT. This together with the excellent safety profile resulted in great patient satisfaction as reflected by 97.5 and 97.1% of patients reporting their intention to use PLUS again and to recommend it to a friend, relative, or colleague, respectively. These data support the suitability of PLUS in the self-management of dysmenorrhea, but future studies are recommended particularly in direct comparison with other self-management options.

## Data Availability

The original contributions presented in the study are included in the article/supplementary material, further inquiries can be directed to the corresponding author.
